# Replication marketplaces would help science to become more self-correcting

**DOI:** 10.1098/rsos.240850

**Published:** 2024-10-02

**Authors:** Joachim Hüffmeier, Clara Kühner

**Affiliations:** ^1^ Institute of Psychology, TU Dortmund University, Dortmund, Germany; ^2^ Wilhelm Wundt Institute of Psychology, Leipzig University, Leipzig, Germany

**Keywords:** replication, scientific self-correction, journal policies, open science, meta-science

## Abstract

Independent replications are very rare in the behavioural and social sciences. This is problematic because they can help to detect ‘false positives’ in published research and, in turn, contribute to scientific self-correction. The lack of replication studies is, among other factors, due to a rather passive editorial approach concerning replications by many journals, which does not encourage and may sometimes even actively discourage submission of replications. In this Perspective article, we advocate for a more proactive editorial approach concerning replications and suggest introducing journal-based *replication marketplaces* as a new publication track. We argue that such replication marketplaces could solve the long-standing problem of lacking independent replications. To establish these marketplaces, a designated part of a journal’s editorial board identifies the most relevant new findings reported within the journal’s pages and publicly offers them for replication. This public offering could be combined with small grants for authors to support these replications. Authors then compete for the first accepted registered report to conduct the related replications and can thus be sure that their replication will be published independent of the later findings. Replication marketplaces would not only increase the prevalence of independent replications but also help science to become more self-correcting.

## 1. Introduction

It is often assumed that science is self-correcting. Mechanisms that are thought to contribute to scientific self-correction include the journal-based peer-review process, published commentaries on recently published research and institutions that are charged with investigating research misconduct such as the Office of Research Integrity [1]. Another major mechanism, with which errors can be detected and corrected, consists in independent replications (i.e., an author [group] B replicates original research by another author [group] A). Especially if such independent replications are conducted and published soon after original findings were published, it would draw attention to potential ‘false positives’ (i.e. studies that find an empirical relationship although no ‘true’ relationship exists). Such false positives can typically not be replicated. Non-replicable findings can be identified based on extant guidelines (e.g. [2]) although there is no broad consensus on the details of how to determine replication success (and failure), and various metrics are used to do so (both multiple frequentist and Bayesian metrics [3]). Original findings that are not replicated in such early, high-quality replications would become the target of further scientific scrutiny, and their influence on later research could be reduced [4].

Despite these potential benefits of replication studies, the prevalence of replications is very low, at least in the behavioural and social sciences [5]. Exemplary estimates show that 0.1% of the published studies for Economics [6] and 1.07% for Psychology are replications [7]. The prevalence has been low for decades, and, if at all, there is only a very slight trend towards more replications across time in some fields [1,8], while the trend may even be regressive in others [9]. Journal policies not encouraging and sometimes even discouraging replications, e.g. by only accepting novel findings for publication [10,11], have kept researchers from conducting replications and represent a substantial publication barrier for the few replications that have been conducted [10]. This passive approach of journal editorship concerning replications is also quantifiable [10]: for instance, in 2022, most peer-reviewed journals in Social Psychology, the psychological discipline arguably most affected by non-replicable findings, did not mention replications at all on their websites (73%), actively discouraged them (2%) or considered them secondary (2%). As another example [11], in 2020, the overwhelming majority of Industrial and Organizational Psychology and Management journals did not mention replications at all on their websites (93.1%) or they considered them secondary (3.5%). Thus, these rather passive or sometimes even defensive approaches to journal editorship concerning replications pose a serious structural problem, at least for behavioural and social sciences disciplines, but possibly also beyond.

If journals simply changed their current approach and decided to encourage replication studies, their prevalence may indeed increase (as shown by prior research [10]). However, the increase would still be too slow for independent replications to function as an effective self-correction mechanism in many scientific disciplines—particularly in those that lack a history of conducting replications by default [5–8]. Moreover, if the selection of original research for replications remains a largely undirected process, important original findings may still never be replicated by independent researchers because replications are often limited to highly cited findings [12]. Finally, various author groups may independently decide to conduct replication studies of particularly prominent findings (e.g. findings published in journals like *Nature* or *Science*), which would result in an unfavourable allocation of limited resources (e.g. time, money, participant and laboratory capacities).

## 2. Measures to increase the number of replications

To increase the number of replications, various measures have been proposed and partly also implemented in different fields. These measures entail, for instance, the explicit invitation by a journal to submit Registered Reports [13] detailing independent replications of findings that were published in that journal—a practice used, for instance, by the psychological flagship journal *Psychological Science*. Although *Psychological Science* is not the only journal using Registered Reports as a measure for further replications, it would be desirable that far more journals take up this measure. The establishment of the so-called Psychological Science Accelerator (i.e. a distributed international network of psychology labs that collaboratively replicates selected original findings [14]) is a further proposed measure to promote replications. This network selects empirical findings and replicates them with large-scale studies that are typically conducted in various involved labs.

A further example consists of journal-based replications (i.e. ‘the publishing journal insists on a replication attempt between acceptance and publication’ [15, p. 1]), which have been tested in a proof-of-concept study [15]. If implemented as a standard, authors would have to replicate their own experimental findings after the (conditional) acceptance of their manuscript, but before it is published. Moreover, it has been suggested that findings that are ‘in the pipeline’ could be replicated in multiple independent laboratories before they are submitted for publication (the ‘pipeline project’ [16]). As a slight variation of this approach, scholars could write a working paper on a new original finding. However, they commit to never publish this working paper. They would then join forces with other scholars (or groups of scholars) interested in the new original finding. These other (groups of) scholars conduct one or several independent replications of this finding [17]. In the final step, the whole group writes up and publishes the new original finding together with all of its replications. Thus, in all latter cases (except for Registered Reports, for instance, in *Psychological Science* and for the Psychological Science Accelerator), the original findings would be published together with the findings from the replications.

We want to state clearly that we highly value all of the above measures and we especially appreciate their collaborative character. We further hope that all these measures will jointly contribute to tackle the current paucity of replications. However, either they are currently restricted to a few journals (e.g., Registered Reports for replications as offered, for instance, by *Psychological Science*), they can only replicate a rather limited number of original findings (e.g. Psychological Science Accelerator), or they have not yet developed beyond the proof-of-concept stage (e.g. journal-based replications or the ‘pipeline project’). Thus, additional ideas are needed that—on their own and together with other measures—may be able to increase the number of published (independent) replications. We want to present such a new idea in the following. Implementation trials could and should test which of the proposed measures—if any—are particularly effective and should be broadly implemented (for details, see below).

## 3. A new measure to promote independent replications: replication marketplaces

To strengthen the capacity of independent replications to function as scientific self-correction mechanisms, we advocate for a proactive approach to journal editorship concerning replications and suggest a new measure that we call *replication marketplaces*. Journals introducing replication marketplaces would offer a new and specific publication track focused exclusively on independent replication studies. To establish a replication marketplace, a designated part of the editorial board (i.e. a replication marketplace committee, for instance, led by the Editor[s]-in-Chief or a designated Replication Editor) would continuously identify new relevant findings from their journal and publicly offer them for replication on the journal’s website.

This selection process should follow a standardized and transparent procedure that considers predefined criteria such as, for instance, the uncertainty of a claim that is made in a study or the utility of a published claim after replication [18]. Claims are, for instance, uncertain if the underlying evidence is ‘sparse and ambiguous’ (see [8, p. 444]), if the applied measures are imprecise, if the employed designs are weak or if there is reason to assume a substantial publication bias [18]. The utility of a published claim refers to the positivity of ‘merely’ generating new or more definite knowledge, substantial theoretical implications, and possible practical applications [18]. In any case, replication marketplace committees would primarily focus on original findings that were published for the first time within their journals’ pages. The goal of a replication marketplace would be to systematically replicate a substantial share of a journal’s original findings (e.g. 20%), and the replication marketplace committee would therefore probably work most efficiently if it issued calls for replications directly with the publication of original findings to ensure timely replication.

Independent, interested authors (i.e. not part of the original research team) could then compete with each other by applying for these offered replications using a registered report format detailing the planned replication study [13]. After receiving a certain number of registered reports (e.g. three) or after a deadline has passed, the journal could close the respective call for replications and inform about this closure on its website to avoid unnecessarily spending researchers’ resources. The first author(s) receiving an in-principal acceptance for their registered report would be allowed to publish it independently of the observed results. In the moment of sending out the in-principal acceptance, the journal would also inform other authors who applied for the same replication and other potentially interested parties on their website that the replication of the study in question is closed because it has been allocated to an applicant (i.e. an author or author group). Authors winning with their bid for an offered replication would then have a fixed amount of time to run the replication study and write it up for publication. If they cannot deliver a manuscript with the results of the agreed replication within the allotted time, the call for a replication of the respective study could be re-opened.

We want to clarify that replication markets should complement (and *not* replace) the possibility of submitting author-initiated registered reports that detail replications of original findings that were first published in a journal and registered reports for new findings. We do not believe that it is wise to restrict the number of high-quality independent replications or ways in which such replications come about. Although a more widespread use of registered reports as a tool to publish original research may eventually make such original research more robust and trustworthy, we believe that independent replication studies will always be needed for scientific self-correction.

## 4. The most effective way to implement replication marketplaces

The new publication track would work most efficiently when implemented with the following features: First, an expedited peer-review process, which would be possible because reviewers would only have to cover the theory and especially the method section of the later article [13]. The expedited review process could be organized by recruiting reviewers primarily from the replication marketplace committee who should be interested in reviewing registered reports from the journal’s replication marketplace. Second, given the very high profits of many academic publishers, it would certainly be desirable that these publishers establish a ‘replication fund’ to support and complement replication marketplaces to facilitate replication studies (i.e. small grants of, for instance, 3.000 € per replication study). While this may not be possible for most journals that are run by scientific societies, publishers such as *Springer Nature*, *Elsevier*, *Wiley*, *Taylor & Francis,* and *Sage* could (and, as we believe, should) spend money on such replication funds. They would thereby take responsibility for the findings they publish and support the scientific community by improving scientific practices and correcting the scientific record.

Third, it is desirable that journals establish a designated replication section. As part of this replication section, a journal would aim to publish replications at a fixed ratio as compared to primary findings (e.g. one replication per five new original findings). That is, if five (10) new findings are published in an issue, the journal would publish one (2) replication studies in the same issue. Fourth, original research that is subject to a later replication could receive a ‘replication batch’ that directs readers’ attention to the replication [19].

Fifth, it is desirable that not only single journals but whole ‘journal families’ of academic publishers (e.g., *Springer Nature*, *Elsevier*, *Wiley*, *Taylor & Francis,* and *Sage*) introduce replication marketplaces. Otherwise, findings published in journals with replication marketplaces would be systematically replicated and therefore become more trustworthy, while findings published in journals without replication marketplaces would still never be replicated and remain less trustworthy. Thus, if only a few journals introduced replication marketplaces, journals without replication marketplaces could even become loopholes for non-replicable and untrustworthy findings. Given that Editors-in-Chief may not always have the authority to change journal policies [11], publishers are often in the best position to introduce replication marketplaces for all of their journals in a given field (i.e. their journal families).

## 5. The advantages and disadvantages of replication marketplaces and other measures

Table 1 provides an overview of the advantages and disadvantages of the various measures that were proposed to promote replications (including replication marketplaces). In the following, we will discuss the specific advantages that replication marketplaces would have compared to other measures: First, journals implementing replication marketplaces would take the arguably *most systematic approach* to replicating original findings because their goal would be to replicate many findings from their own journal. Thus, if replication marketplace committees continuously select the studies that are most worthy of replications (e.g. in view of the uncertainty and utility of their published claims [18]), they can determine how replicable and robust research in their journals’ pages is. They can also contribute to a more successful cumulative science because researchers would less often build on original research that—often much later (e.g. [20,21])—turns out to be rather difficult to replicate.

**Table 1.** Overview of advantages and disadvantages of proposed measures to further replications.

	Registered Reports for independent replications (e.g. as offered by *Psychological Science*)	*Psychological Science Accelerator*	journal-based replications	Large-scale replication projects modelled after the ‘Pipeline Project’	replications with other authors based on a new original finding and a working paper	replication marketplaces
Advantages	well-established measure with high methodological quality due to peer review prior to data collection collaborative approach (if multiple author teams are involved) pottery barn rule is considered submission may be based on the detection of flaws by expert researchers	well-established measure collaborative approach submission may be based on the detection of flaws by expert researchers large-scale, international replication studies with high statistical power and the possibility of testing numerous moderators/boundary conditions	promising measure technically easy to implement as a standard approach pottery barn rule is considered journals could determine the replicability of their findings (if established as a standard for a journal)	promising measure collaborative approach large- scale, international replication studies with high statistical power and the possibility of testing numerous moderators/boundary conditions	promising measure collaborative approach pottery barn rule is considered (if established as a standard for a journal)	directed process and systematic approach: can ensure replication of many findings choice of studies to be replicated based on clear criteria measure with high methodological quality due to peer review prior to data collection transparent: would avoid coordination problems between author groups pottery barn rule is considered Incentive for authors to conduct replications
Disadvantages	undirected process and unsystematic approach: registered reports containing replications may be submitted more or less regularly and can thus not ensure that many published findings are replicated	limited capacity: only few studies are replicated with this approach unsystematic approach: cannot ensure that many published findings are replicated	in the proof-of-concept stage no independent replications (i.e., the same author team) costly for the authors (financial costs) unclear, whether authors would agree to the procedure or simply publish in another journal mostly (only?) suited for experiments	in the proof-of-concept stage costly for the authors (costs for finding replication partners) limited capacity: only few studies can be replicated with this approach unsystematic approach: cannot ensure that many published findings are replicated	in the proof-of-concept stage costly for the authors (costs for finding replication partners) mostly (only?) suited for experiments	in the idea stage (not tested yet) costs: some registered reports would potentially be rejected per specific call for replication competition could lead to unethical behaviour (e.g., researchers take shortcuts in preparing registered reports)

Second, they would do so in a regular and criteria-based way. Thus, journals that implement replication marketplaces would issue regular calls for replications and thereby take a lot of responsibility for novel claims that are published for the first time in their pages (i.e. a pottery barn rule for scientific journals [22]). While some other approaches would also consider the pottery barn rule (see, for instance, the possibility to publish replications in *Psychological Science* or *Royal Society Open Science*), replication marketplaces would do so in the most systematic way. Third, replication marketplaces would be a very transparent measure that avoids coordination problems between different (groups of) researchers. For instance, issued calls for specific replications would clearly communicate which original findings should be replicated, the call would be closed after a certain number of registered reports are submitted or a deadline has passed, and the acceptance of Stage 1 Registered Reports would also be published. These measures contribute to avoiding that different (groups of) researchers unknowingly set out to replicate the same original finding.

Fourth, and this would be an interesting byproduct of replication marketplaces, they could alleviate the underrepresentation of researchers from middle- and low-income countries. Thus far, researchers from such countries are drastically underrepresented as authors, especially in the most prestigious journals of a discipline [23]. The possibility of participating in replication marketplaces and submitting a convincing registered report may make it easier than ever for them to publish in these journals. This is because the highly standardized format of registered reports [13], especially for replication studies, and a double-blind review procedure may give away less personalized information about the authors than full manuscripts with original research. The lower availability of personalized information may then also offer fewer opportunities for discriminating against researchers from middle- and low-income countries in the peer-review process. If journals complement their replication marketplace with a ‘replication fund’ to facilitate replication studies, they could additionally remove lacking financial resources as another barrier for these researchers.

Replication marketplaces, of course, also have distinct disadvantages that other measures do not have. First, in contrast to the other measures described above, replication marketplaces currently have the status of a mere idea. Thus, it is not yet clear whether they can, in fact, be effective in promoting independent replications. Second, if several (groups of) authors compete for the first accepted registered report, some registered reports are eventually rejected, and the resources invested in these registered reports are (mostly) wasted. Third, the competition that would be involved in replication marketplaces could result in unethical behaviour. Relevant unethical behaviours would consist of researchers taking shortcuts in preparing the registered report just to be first (e.g. omitting necessary piloting of materials and/or procedures). Possibly, however, not all such unethical behaviours necessarily have problematic consequences, because at least part of these omissions could be detected during the review process.

## 6. Testing the feasibility and effectiveness of replication marketplaces

Measures to promote independent replications should only be implemented broadly if their feasibility and effectiveness are well established. Thus, we suggest that, in a first step, the feasibility of replication marketplaces should be tested by implementing such a marketplace at a first journal for a limited time span (e.g. 3 years). If a replication marketplace can be successfully implemented and maintained with a justifiable investment of resources and if it can in fact increase the number of published replications in the related journal, the second step should follow. In this step, it should be tested whether the expected effect of implementing replication marketplaces on the number of published replications goes beyond and adds to the effect that other measures offered by some journals already have (e.g. author-initiated registered reports for replications at *Psychological Science*). It should also be tested whether possible negative effects of replication marketplaces outweigh their expected positive effects. If the second step is also successful, a large-scale implementation of replication marketplaces may be recommendable.

## 7. Conclusion

In sum, if many journals in a field decided to implement replication marketplaces, these marketplaces could (i) increase the chance that the most important original findings in a field would be regularly tested with independent replications, (ii) offer authors a strong incentive to conduct independent replications, (iii) represent a systematic track to publish them, thereby (iv) strengthening scientific self-correction in many scientific disciplines while avoiding undirected (and sometimes even unnecessary) and resource-intensive replication efforts. On the other hand, the idea of replication marketplaces has not been empirically tested yet. Thus, it is not clear (i) whether replication marketplaces would be feasible with an investment of limited resources, (ii) whether the expected positive effects would in fact emerge, and (iii) whether and to which degree they would outweigh possible negative effects. To assess the potential of replication marketplaces and to answer these questions, we suggest that future research may start with a first proof-of-concept study. If this study is successful, further studies could test replication marketplaces’ effectiveness in combination with and against other measures that are thought to promote independent replications.

## Data Availability

This article has no additional data.
